# Determining Sufficient Number of Imputations Using Variance of Imputation Variances: Data from 2012 NAMCS Physician Workflow Mail Survey^*^

**DOI:** 10.4236/am.2014.521319

**Published:** 2014-12

**Authors:** Qiyuan Pan, Rong Wei, Iris Shimizu, Eric Jamoom

**Affiliations:** National Center for Health Statistics, Centers for Disease Control and Prevention, Hyattsville, MD, USA

**Keywords:** Multiple Imputation, Sufficient Number of Imputations, Hot-Deck Imputation

## Abstract

How many imputations are sufficient in multiple imputations? The answer given by different researchers varies from as few as 2 - 3 to as many as hundreds. Perhaps no single number of imputations would fit all situations. In this study, *η*, the minimally sufficient number of imputations, was determined based on the relationship between *m*, the number of imputations, and *ω*, the standard error of imputation variances using the 2012 National Ambulatory Medical Care Survey (NAMCS) Physician Workflow mail survey. Five variables of various value ranges, variances, and missing data percentages were tested. For all variables tested, *ω* decreased as *m* increased. The *m* value above which the cost of further increase in *m* would outweigh the benefit of reducing *ω* was recognized as the *η*. This method has a potential to be used by anyone to determine *η* that fits his or her own data situation.

## 1. Introduction

As a way of handling missing data, multiple imputation (MI) has been steadily gaining popularity in the past several decades. When it comes to the question of how many imputations are sufficient in applying MI, different researchers have given different answers. Rubin suggested 2 to 5 [1] [2]. Schafer and Olsen suggested 3 to 5 [3]. Graham *et al.* suggested 20 or more [4]. Hershberger and Fisher suggested that several hundred imputations are often required [5]. Allison suggested that one may need more imputations than what were generally recommended in the literature [6]. The primary reason for the variation in recommended numbers of imputations is that different researchers may use different parameters or statistics and what is good for one parameter or statistic may not be good enough for a different parameter or statistic. For example, Rubin used the “relative efficiency” as defined by him to make the decision [1], whereas Graham *et al.* used the testing power to make the decision [4]. When a data user decides to adopt MI, he or she does not have standard guidelines on how many imputations he or she should use for his or her particular data situation.

Variables that need imputation can be different, and the imputation models and/or methods can vary. The resources that can be allocated to MI application can also vary from survey to survey. It may not be practical to recommend a specific number of imputations that can universally fit all imputation models or data and management situations. Instead of attempting to prove or disprove recommendations found in the published literature, the current research examines an empirically-based method to determine the minimally sufficient number of imputations needed.

Let *m* be the number of imputations and *η* be the least number of imputations that is sufficient to meet a minimum requirement chosen by a data analyst. Among the various published answers to the “how-many-are-sufficient” question, the one given by Rubin (1987) [1] appears to be by far the most influential. In Chapter 4 of his classic book on MI, Rubin established that the relationship between *V*(*Q_m_*), the large sample variance of a point estimator, *Q*, from a finite *m*, and (*Q_∞_*), the variance from an infinite *m*, is

(1) $$V\left(Q_{m}\right)=\left(1+\frac{\gamma_{0}}{m}\right)V\left(Q_{\infty }\right),$$

where *γ*_0_ is the population fraction of missing information. From this relationship, the relative efficiency (RE) measured in the units of standard errors from using MI is

(2) $$\mathrm{RE}={\left(1+\frac{\gamma_{0}}{m}\right)}^{-1∕2}.$$

Based on this RE, Rubin stated: “If *γ*_0_ ≤ 0.2, even two repeated imputations appear to result in accurate levels, and three repeated imputations result in accurate levels even when *γ*_0_ = 0.5”.

The question is: Is the RE, as defined by Equation (2), the best and only parameter that can be used as the criterion to determine *η*? Different *η* recommendations from different researchers (e.g. [3]-[5]) suggest this RE may not be the best and only parameter to determine *η*. None of these researchers including Rubin have explicitly claimed that his or her method is the only correct method to determine *η*.

The number of imputations affects the MI results in multiple ways as indicated by the following equations for MI data analyses [1]. The mean of the point estimator *Q* is

(3) $$\bar{Q}=\frac{1}{m}\sum_{1}^{m}Q_{i}.$$

The average variance estimate of *Q* over *m* complete datasets from MI is

(4) $$\bar{U}=\frac{1}{m}\sum_{1}^{m}U_{i},$$

where *U_i_* is the variance estimate of the *i*th imputation. The estimated imputation variance is

(5) $$B=\frac{1}{m-1}\sum_{1}^{m}{\left(Q_{i}-\bar{Q}\right)}^{2}.$$

The total variance estimate is

(6) $$T=\bar{U}+\left(1+m^{-1}\right)B.$$

Equations (3) to (6) all contain *m* as a factor, indicating that *m* can affect the results of MI data analyses in multiple ways. The minimally sufficient number of imputations could be defined as the smallest *m* that would produce a sufficiently accurate *Q̄*, *Ū*, *B*, or *T* as judged by the data user.

This paper presents a methodology to determine *η* by focusing on the effects of *m* on the accuracy of *B* as defined by Equation (5). The focus is on *B* because the primary advantage of MI over single imputation (SI) is that MI makes it possible to estimate *B* while SI cannot [1] [7]. For an *m* to become the *η*, this *m* should first be capable of allowing us to obtain a sufficiently accurate *B*. The accuracy of *B* can be measured by *ω*, the estimated standard error of *B*. For a particular variable, *ω* would be smaller as *m* increases. If *ω* becomes sufficiently small, then one may conclude that *B* is sufficiently accurate and the *m* corresponding to that *ω* value would represent *η*. Using the data from the 2012 wave of the National Ambulatory Medical Care Survey (NAMCS) Physician Workflow study (PWS), we were able to demonstrate that *ω* can be used to determine *η*. This method can be used by anyone to determine *η* for his or her own data.

## 2. Methodology

### 2.1. The Survey

The PWS is a nationally representative, 3-year panel mail survey of office-based physicians conducted by the National Center for Health Statistics [8]. The PWS sample includes 5266 physicians who were confirmed eligible in the 2011 Electronic Medical Records mail survey, a supplement to the NAMCS. To meet eligibility criteria, physicians had to see ambulatory patients in office-based settings. All eligible physicians were mailed the first wave of the study in 2011. Sampled physicians who did not respond to the first mailing were sent up to two additional mailings, and survey administrators followed up by phone with those who did not respond to any of the three mailings. A total of 3180 eligible physicians responded in the 2011 PWS, yielding a weighted response rate of 46.0 percent. All eligible physicians, including those who did not respond in 2011, were mailed a second survey in 2012. This 2012 cycle of data yielded 2567 eligible responses, for a weighted response rate of 42.1 percent and was used for the current MI research. Missing value percentages were calculated by regarding the 2567 records as the complete data set and the imputations were carried out within these 2567 records. All analyses in this research were conducted with unweighted data.

### 2.2. The Imputed Variables

When resources permit, it is desirable to include more variables of different characteristics to make the conclu- sions from the study have better reference values to other data users and researchers. The variables selected for imputations in this study are described in Table 1. Five variables were selected from the survey for MI tests. PRACSIZE2, PRACSIZE5, PRACSIZE20, and PRACSIZE100 represent the number of physicians in the practice. PRACTSIZE100 is the original variable with values ranging from 1 to 100. PRACSIZE2 and PRACSIZE5 were derived from PRACTSIZE100 by recoding the values into 2 and 5 categories, respectively, and PRACSIZE20 was derived from PRACTSIZE100 by top-coding the >20 values to 20. These four variables had The same percent of missing data of 3.62% but different value ranges and variances. The fifth variable, CLSTAFF1, was the number of clinical staff. It had a value range of 0 - 99, which was similar to that of PRACTSIZE100, but a missing data percentage of 8.88%, which was different from that of the other four variables.

### 2.3. The Imputations

Hot deck imputation [9] was used in this MI study. The donor groups for the hot deck imputation were defined by the region of the physician's interview office (REGION), the physician's interview specialty group (SPECR), and the physician's primary present employment code (PRIMEMP) (Table 1). These three variables were chosen for defining the donor groups in order to minimize possible correlation between the donor groups and the variables to be imputed. The donors for missing values were randomly selected with replacement from the donor group which matched the recipient. A total of 500 imputations were obtained for each variable. The pool of 500 imputations was used as the population and a random sample of *m* imputations was drawn from the pool for the MI.

### 2.4. Determination of the Variance of the Imputation Variances

**Figure 1:**
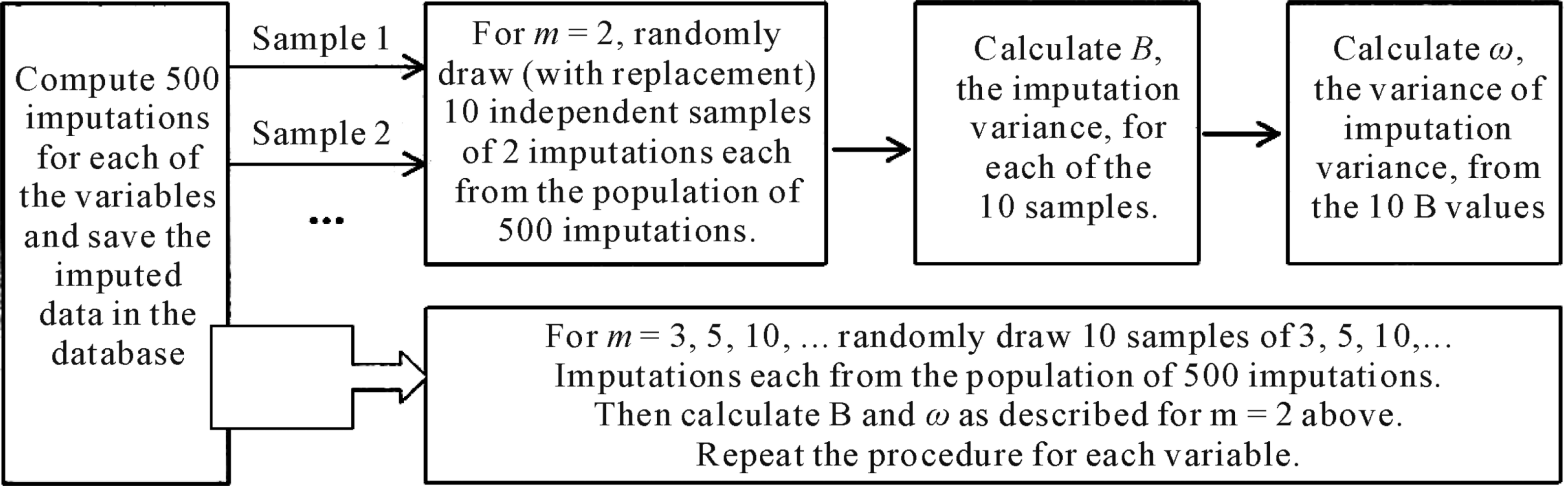
Experimental design for obtaining *B*, the imputation variance, and *ω*, the standard error of *B*, for different *m*, the number of imputations.

Thirteen different numbers of imputations were tested for MI: *m* = 2, 3, 5, 10, 15, 20, 25, 30, 35, 40, 60, 80, and 100. The imputation variance (*B*), defined by Equation (5), was calculated for the data obtained from each MI. In order to calculate the variance of *B*, 10 independent random samples from the 500 imputations were pulled for each *m* of each variable. Figure 1 describes the process in a diagram. *V_B_*, the variance of *B*, is estimated by Equation (7):

(7) $$V_{B}=\frac{1}{\left(n-1\right)}\sum_{1}^{n}{\left(B-\bar{B}\right)}^{2},$$

where *n* is the number of MI samples for a given *m*, which is 10 for the current study. The standard deviation of *B* is:

(8) $${\mathrm{SD}}_{B}=\sqrt{V_{B}}=\sqrt{\frac{1}{\left(n-1\right)}\sum_{1}^{n}{\left(B-\bar{B}\right)}^{2}}.$$

The standard error of *B* was calculated using the following formula:

(9) $$\omega =\sqrt{\frac{V_{B}}{n}}=\sqrt{\frac{1}{n\left(n-1\right)}\sum_{1}^{n}{\left(B-\bar{B}\right)}^{2}},$$

*V_B_*, SD*_B_*, and *ω* measure the variance of imputation variances at different scales.

### 2.5. Determination of Sufficient Number of Imputations (*η*)

For a given *m-ω* curve, different data users may have different criteria for a particular *m* value to be recognized as being “sufficient” and so arrive at with different *η* values. Two methods were developed to determine *η*, one is called “the moving regression method” and the other is called “the confidence interval method”. These two methods do not exhaust the possibilities of other ways for using the *m-ω* relationship to determine *η*.

#### 2.5.1. The Moving Regression Method

The linear model *Y* = *βX* + *a* was used to fit the *m-ω* relationship in a “moving” fashion, where the independent variable *X* is *m* and the dependent variable *Y* is *ω*. Let *X*_1_,*X*_2_,*X*_3_,···,*X_j_* be the values of the independent variable *m* arranging from the smallest to the largest and *Y*_1_,*Y*_2_,*Y*_3_,···,*Y_j_* be the corresponding values of *ω*. Let *h* be the number of independent variable values included in each regression group. Fit the regression model for *m* = *X*_1_,*X*_2_,*X*_3_,···,*X_h_*, then for *m* = *X*_2_,*X*_3_,*X*_4_,···,*X*_*h*+1_, then for *m* = *X*_3_,*X*_4_,*X*_5_,···,*X*_*h*+3_ and so on. The relative slope for 5 – *m* increment (*RS*_5_) is derived and a cutoff point is chosen for *RS*_5_ to determine *η*. *RS*_5_ is defined as:

(10) $$RS_{5}=100\frac{S_{5}}{\bar{\omega }}=100\frac{S_{5}}{\frac{1}{h}\sum_{1}^{h}\omega },$$

where *S*_5_ is the slope of the regression line using an increment of five in *m* as a unit of *X*, and $\bar{\omega }$ is the mean of *ω* in the particular regression group. The *S*_5_ is the standardized *ω* reduction slope for every 5-imputation increase in *m*. *RS*_5_ is the percentage of *S*_5_ over $\bar{\omega }$. The worksheet for variable PRACSIZE5 is given in Table 2 to illustrate this method.

Within the range of *m* where *m* has a detectable effect on *ω*, *RS*_5_ should be negative, and the absolute value of *RS*_5_ (|*RS*_5_|) should decrease with the increase in *m*. With only three data points included in a regression, however, a single outlier of *ω* may result in outlier *RS*_5_ values. Suppose |*RS*_5_| = 10 is the cutoff point to determine *η*. An |*RS*_5_| is called an outlier if it satisfies the following two conditions: 1) It is less than the cutoff point; 2) There are |*RS*_5_| values that are greater than the cutoff point within the next three *RS*_5_ values upstream, *i.e.* in the direction of higher *m*.

Going from the lowest *m* to the highest *m*, if there are no outliers, the middle point *m* of the first regression with |*RS*_5_| < 10 would be the *η*. In the current case, an outlier *ω* at *m* = 25 (Table 2) has resulted in two outlier *RS*_5_ values, −1.03 (from the regression of *m* = 15, 20, 25) and −9.03 (from the regression of *m* = 20, 25, 30). The following option is suggested to deal with outliers: Let *m* be the *m* corresponding to the outlier *ω*. Take the average of the two *RS*_5_ values whose corresponding regressions have *om* as their largest and smallest *m*, respectively. If the average |*RS*_5_| is less than the cutoff point, then the *om* would be the *η*. If the average is |*RS*_5_| is greater than the cutoff point, then all the |*RS*_5_| values related to the outlier *ω* are treated as being greater than the cutoff point. In the current case, the two regressions with the *om* as their largest and the smallest *m* have *m* = 15, 20, 25 and *m* = 25, 30, 35 and the corresponding *RS*_5_ values −1.03 and −27.79, respectively. The average |*RS*_5_| is 14.41 > 10. Therefore, neither −1.03 nor −9.03 would be recognized as the first |*RS*_5_| < 10 values. The regression of the first |*RS*_5_| < 10 has *m* = 40, 60, 80. Therefore, the *η* is determined to be 60 for PRACTSIZE5 using this method.

#### 2.5.2. The Confidence Interval Method

In the confidence interval method, *ω* is used to calculate the 95% confidence interval for *B̄*, the mean of the 10 *B* values of each *m*. Because *B̄* is the sample mean of *B* which is a chi-square variable with *m* – 1 degrees of freedom, the normal distribution can be used to approximate the distribution of *B̄* for sufficiently large values of *m* [10]. For PRACTSIZES, *m* = 2 was verified to be sufficiently large for *B̄* to approach normality (not shown). As a result, for a sample size of 10, the Student *t* test can be used to calculate the confidence interval of *B̄*. Then *P*, the percentage of half-width of the confidence interval divided by *B̄* is derived and used as the parameter to determine *η*. *P* is defined by the following equation:

(11) $$P=100\left(t_{0.05}\omega \right)∕\bar{B},$$

where *t*_0.05_ is the *t* value from the Student *t* test at 0.05 probability level and the term “*t*_0.05_*ω*” represents half the width of the 95% confidence interval because the full width of the 95% confidence interval would be $\left[\bar{B}+\left(t_{0.05}\omega \right)\right]\left[\bar{B}-\left(t_{0.05}\omega \right)\right]=2t_{0.05}\omega$. The variable PRACSIZE5 was used to illustrate this method and the results are presented in Table 3. Data in Table 3 show that P decreased as *m* increased. It is up to each data analyst to decide the cut-off point at which the *m* would be recognized as sufficiently large so that this *m* would become *η*. The minimum *m* value above the chosen cutoff point of *P* would be the *η*. For illustration, then *η* is determined to be 40 for PRACTSIZE5 if *P* = 15 is chosen as the cutoff point.

## 3. Results

### 3.1. Effects of *m* on *B*

The magnitude of *B*, the imputation variance as defined by Equation (5), was different among the five variables (Table 4). For example, at *m* = 100, the mean of the *B* was 0.101, 1.02, 15.6, 186, and 232 for variables PRACSIZE2, PRACSIZE5, PRACSIZE20, PRACSIZE100, and CLSTAFF1, respectively (Table 4). The difference in *B* values reflected the huge difference in the variances among the five variables. Within each variable, the mean of *B* did not show a clear trend of increase or decrease as *m* was increased from 2 to 100 (Table 4). This was expected because the sample mean of *B* would fluctuate around the population *B* value, which should not be affected by the changes in *m*.

**Figure 2:**
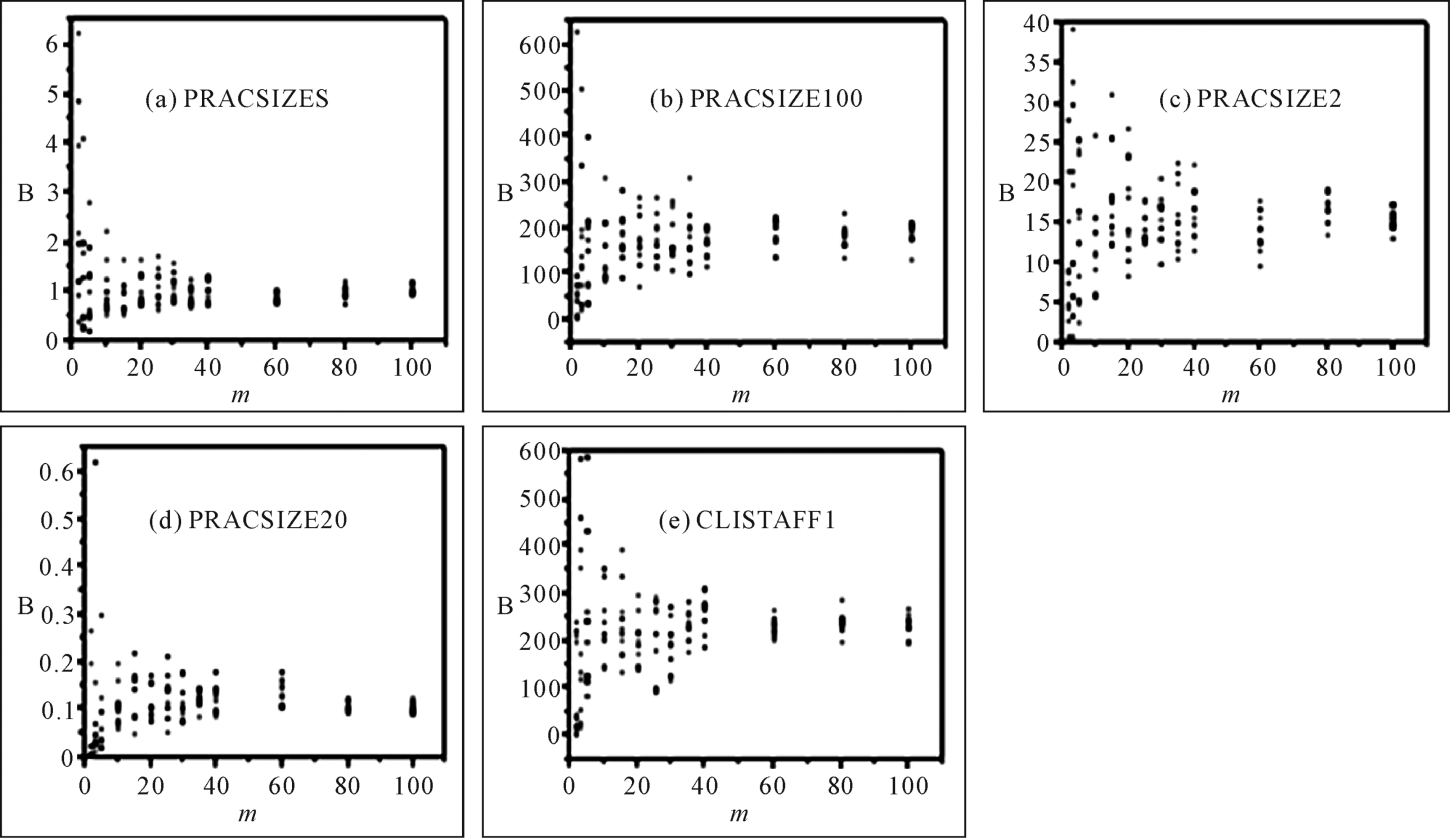
Effects of number of imputations (*m*) on imputation variances (*B*).

Individual *B* values at each *m* were plotted in Figure 2. Within each variable, the individual *B* values were scattered across a wide range when *m* was small (<20) (Figure 2). For example, for variable PRACSIZE5 at *m* = 3, the *B* value was 0.2186 from one sample, and 4.0896 from another sample. This means that if one decided to use *m* = 3 for MI, the imputation variance could by chance be many times bigger or smaller than the true *B* value. The widely scattered data points at low *m* values in Figure 2 indicate that when *m* was less than 20 or even 40, the *B* value obtained may not be accurate and reliable.

### 3.2. Effect of *m* on *ω*

**Figure 3:**
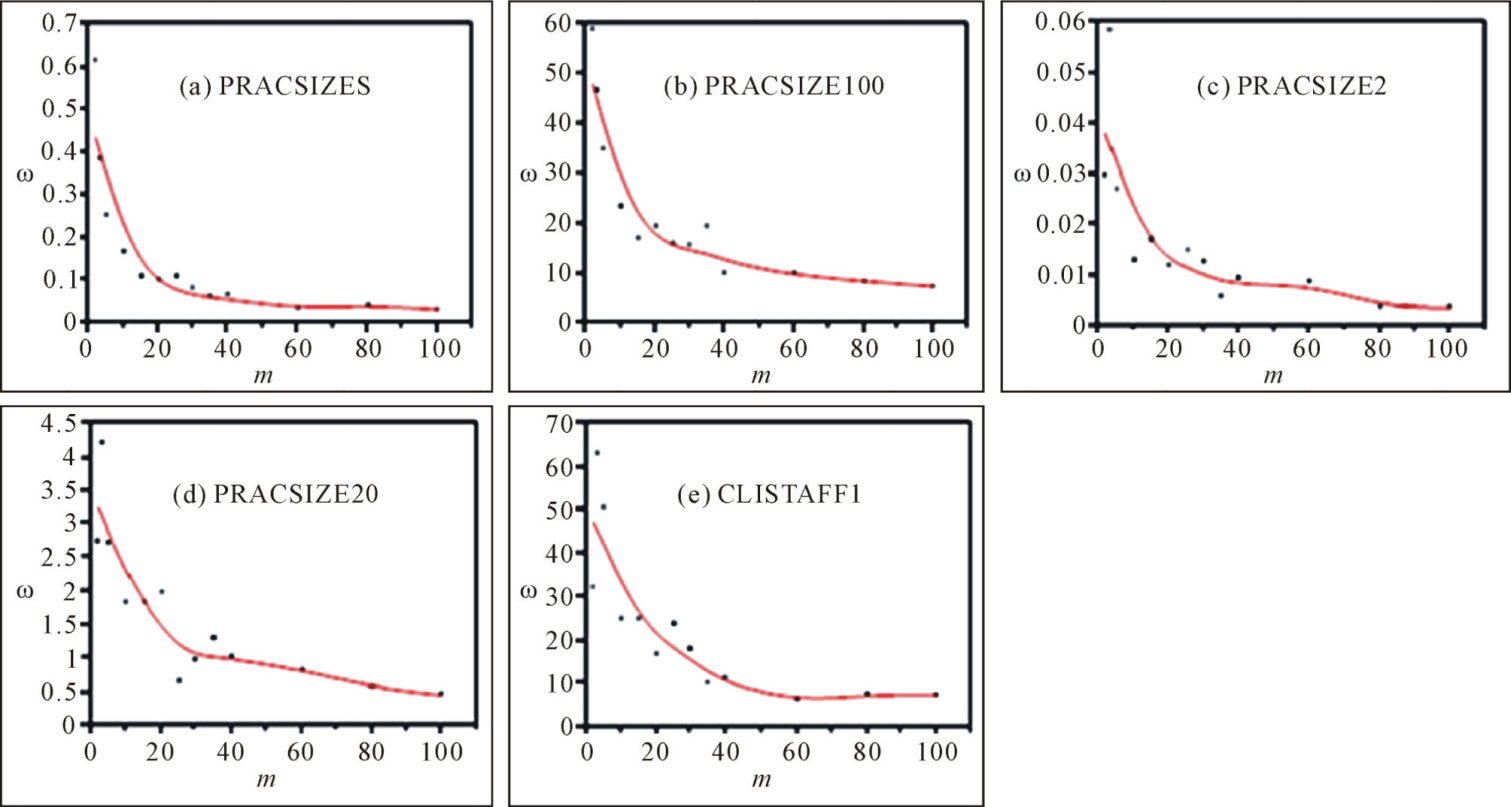
Effects of number of imputations (*m*) on the variance of the imputation variances (*ω*).

Figure 3 presents data on *ω* for different *m* values. Measured in standard error, *ω* was the largest when *m* = 2 or 3. It quickly decreased with the increase in *m* and tended to stabilize as *m* approached 100, the highest *m* tested. Taking the variable PRACSIZE5 as an example, *ω* decreased by 82% when *m* increased from 2 to 15, then decreased an additional 13% when *m* increased from 15 to 100. The *m-ω* curve was similar among all the five variables tested even though these variables were different in their value range and other characteristics (Figure 3; Table 1).

### 3.3. Sufficient Number of Imputations

Sufficient numbers of imputations (*η*) as determined by the moving regression method (see Section 2.5.1) and the confidence interval method (see Section 2.5.2) for the five variables tested are listed in Table 5. The two methods often gave different *η* values for the same variable from the same *m-ω* relationship. To be specific, the moving regression method resulted in an *η* of 30 to 80, whereas the confidence interval method resulted in an *η* of 25 to 40 (Table 5). These results suggest that the decision on criteria for what should be considered “sufficient” plays an important role in determining *η*.

## 4. Discussions

Shifting from SI to MI allows an estimate of *B*, the imputation variance, and a more accurate estimate of the total variance, *T*, as defined by Equation (6) [1] [7]. If an MI protocol cannot give a reliable estimate of *B*, then the major benefit of MI would be lost. Therefore, it makes sense to use an *m*-dependent measure of reliability for the estimate of *B* as a criterion in determining *η*, the minimally sufficient number of imputations. The results of this research indicate that *B* values obtained for *m* < 20 may not be reliable at least for these five variables tested using the imputation method described (Figure 2 and Figure 3). If one uses Rubin's recommendation of *m* = 2 or 3, the *B* value obtained may be many times bigger or smaller than the true *B* value. Using the criterion of whether a reliable *B* can be obtained using the protocol described in this paper, the recommended *m* = 2 to 5 ([1] [2]) are not sufficient for the five variables tested.

The variance of imputation variances is a good determiner of *η* for three reasons. First, it is critical for any MI procedure to produce a reliable estimate of *B*. Second, the effect of *m* on *ω* can be big and can be easily visualized, as shown in Figure 3. Furthermore, the effect of *m* on *ω* decreases with increased *m*, allowing the data user to set a cutoff point for an *m* to be recognized as the *η*. Third, the method of calculating *ω* is relatively simple and practical.

To determine the minimally sufficient number of imputations, one has to first define the criteria for being recognized as “sufficient”. Different data users may have different criteria. As a result, different *η* values may be obtained from the same *m-ω* relationship data, as exemplified by the results show in Table 5 for the two different *η* determination methods discussed above. This is another reason why a universal recommendation of *η* is not a good idea and may even be impossible.

The highest *m* value tested in this study was 100. There was an indication that even *m* = 100 still had some effect in reducing *ω* (Figure 3(b), Figure 3(c) and Figure 3(d)). Ten samples were pulled for each *m.* If one pulled 100 samples for each *m* and tested *m* values of 200, 300, or even greater, it may be possible to show that an increase in *m* would still be statistically effective in reducing the variance of *B*. Theoretically the effects of *m* in reducing *ω* would approach zero but may never become zero. Therefore, the argument by some researchers ([5]) that *η* should be in hundreds is not totally unreasonable and has some support from this research. But because an increased *m* means a higher cost, it may not be justified to do many more imputations just for a tiny bit of gain. Balance of the gain against the cost based on one's own situation plays an important role in η determination.

Based on discussions above, *η* can be defined as the value of *m* beyond which the cost of further increase in *m* would outweigh the benefit of reducing *ω*. Both the moving regression method and the confidence interval method were used to determine *η* based on the *m-ω* relationships obtained for five survey variables. The two methods are meant to be examples to illustrate that different *η* values may be obtained depending on the chosen method and the cutoff point. There may be other methods to determine *η* from a *m-ω* curve that fit one's particular situation better. With the ever-improving information technology, the cost for increased number of imputations may not be a major source of cost in determining *η*. But there is always a need to consider one's particular situation such as one's tolerance level for the probability in determining *η*.

The five variables are different in their value ranges, variances and missing data percentages. Nevertheless the *m-ω* relationship curve can be used to determine *η* for all of the five variables tested. No obvious relationship can be established between the variable characteristics such as the variances and the *η* values obtained.

## 5. Conclusions

### 5.1. The Variance of Imputation Variances Is a Good Determiner for Data—Specific *η*

The primary advantage of MI over SI is that MI makes it possible to obtain an estimate of *B* while SI cannot. Ideally *m* should be large enough to ensure a reliable estimate of *B*, otherwise the MI advantage would be compromised. The reliability of *B* can be measured by *ω*. In this study, the *ω* decreases as *m* increases and the unit gain from increased *m* decreases with greater *m.* These characteristics of the *m-ω* curve make *ω* an ideal determiner of *η*. The method described in this paper can be used by any data user to determine the *η* that fits his or her particular data situation.

### 5.2. Sufficient Number of Imputations Is Larger Than Popular Recommendations

The most popular recommendation for *η* is between 2 and 5, suggested by Rubin [1] [2]. Our results indicate that 2 - 5, or even 10, imputations may not be sufficient to obtain a statistically reliable *B* in MI data analyses at least for the five survey variables tested in this research.

## Figures and Tables

**Table 1:** Characteristics of variables used.

Variable name	Description	Possible values	Missing (%)
The imputed variables			
PRACSIZE2	Practice size grouped into 2 groups	1, 2	3.62
PRACSIZE5	Practice size grouped into 5 groups	1, 2, 3, 4, 5	3.62
PRACSIZE20	Practice size top-coded to20	1 to 20	3.62
PRACSIZE100	Practice size: The number of physicians at the reporting location	1 to 100	3.62
CLSTAFF1	Number of clinical staff	0 to 99	8.88
	The independent variable used in hot-deck imputation		
REGION	Region of the physician's interview office	1, 2, 3, 4	0
SPECR	Physician specialty	1, 3, 4, 5, ···, 15	0
PRIMEMP	Primary present employment code	11, 13, 20, 21, 22, 23, 30, 31	0

**Table 2:** The worksheet of the moving regression method (linear model: *ω* = *βm* + *a*) for determining the minimum sufficient number of imputations (*η*) using PRACSIZE5 as an example.

*m*	* ω *	*S*_5_^a^	$\bar{\omega }$ ^b^	*RS*_5_^c^
2, 3, 5	0.6127, 0.3839, 0.2542	–0.5585	0.4169	–133.95
3, 5, 10	0.3839, 0.2542, 0.1660	–0.1425	0.2680	–53.17
5, 10, 15	0.2542, 0.1660, 0.1100	–0.0721	0.1767	–40.80
10, 15, 20	0.1660, 0.1100, 0.1011	–0.0325	0.1257	–25.86
15, 20, 25	0.1100, 0.1011, 0.1079	–0.0011	0.1063	–1.03
20, 25, 30	0.1011, 0.1079, 0.0835	–0.0088	0.0975	–9.03
25, 30, 35	0.1079, 0.0835, 0.0612	–0.0234	0.0842	–27.79
30, 35, 40	0.0835, 0.0612, 0.0666	–0.0085	0.0704	–12.07
35, 40, 60	0.0612, 0.0666, 0.0342	–0.0060	0.0540	–11.11
40, 60, 80	0.0666, 0.0342, 0.0420	–0.0031	0.0476	–6.46
60, 80, 100	0.0342, 0.042, 0.0311	–0.0004	0.0358	–1.05

a The standardized regression slope using an increment of 5 in *m* as a unit

b The mean of *ω* values included in the regression

c The percentage of *S*_5_ divided by $\bar{\omega }$.

**Table 3:** The worksheet of the confidence interval method for determination of the sufficient number of imputations (*η*) using PRACSIZE5 as an example.

*m*	*B̄*	* ω *	Half confidence interval (*t*_0.05_ * *ω*)	Percentage of half confidence interval over *B̄*(100 * *t*_0.05_ * *ω/B̄*)
2	2.395	0.613	1.386	57.875
3	1.290	0.384	0.868	67.326
5	1.048	0.254	0.575	54.840
10	1.030	0.166	0.375	36.446
15	0.876	0.110	0.249	28.400
20	0.979	0.101	0.229	23.360
25	1.085	0.108	0.244	22.495
30	1.084	0.083	0.189	17.422
35	0.912	0.061	0.138	15.176
40	1.033	0.067	0.151	14.582
60	0.864	0.034	0.077	8.951
80	0.964	0.042	0.095	9.858
100	1.024	0.031	0.070	6.876

^a^ *B̄* = The mean of *B* (imputation variance).

**Table 4:** The effects of mean of the imputation variances from the 10 samples at different number of imputations (*m*).

Variable	*m*							
	2	10	20	30	40	60	80	100
PRACSIZE2	0.053	0.108	0.116	0.107	0.120	0.131	0.107	0.101
PRACSIZE5	2.39	1.03	0.98	1.08	1.03	0.86	0.96	1.02
PRACSIZE20	10.2	12.8	16.8	15.4	16.2	14.1	16.6	15.6
PRACSIZE100	103	158	168	171	168	184	178	186
CLSTAFF1	100	244	197	200	255	228	238	232

**Table 5:** Sufficient numbers of imputations (*η*) as determined by both the moving regression method (Section 2.5.1) and the confidence interval method (see Section 2.5.2).

Variable	*η* from moving regression method	*η* from confidence interval method
PRACSIZE2	60	35
PRACSIZE5	60	40
PRACSIZE20	30	25
PRACSIZE100	40	40
CLSTAFF1	80	35
